# How do older people describe their sensory experiences of the natural world? A systematic review of the qualitative evidence

**DOI:** 10.1186/s12877-016-0288-0

**Published:** 2016-06-01

**Authors:** Noreen Orr, Alexandra Wagstaffe, Simon Briscoe, Ruth Garside

**Affiliations:** European Centre for Environment and Human Health, University of Exeter Medical School, University of Exeter, Knowledge Spa, Truro, UK; The Sensory Trust, c/o Eden Project, Bodelva, Cornwall, UK; PenCLAHRC, University of Exeter Medical School, University of Exeter, Exeter, UK

**Keywords:** Nature, Qualitative, Sensory, Older people, Environment, Outdoors, Systematic review

## Abstract

**Background:**

Despite the increased scholarly interest in the senses and sensory experiences, the topic of older people’s sensory engagement with nature is currently under researched. This paper reviews and synthesises qualitative research evidence about how older people, including those living with dementia, describe their sensory engagement with the natural world.

**Methods:**

Ten databases were searched from 1990 to September 2014: MEDLINE (Ovid), MEDLINE-in-Process (Ovid), PsycINFO (Ovid), CINAHL (EBSCO), GreenFILE (EBSCO), ProQuest Sociology, ASSIA (ProQuest), International Bibliography of the Social Sciences (ProQuest); HMIC (Ovid); Social Policy and Practice (Ovid). Forward and backward citation chasing of included articles was conducted; 20 organizations were contacted to identify unpublished reports. Screening was undertaken independently by two reviewers.

**Results:**

Twenty seven studies were included*.* Thematic analysis revealed that descriptions of sensory experiences are encompassed within six themes: descriptions from ‘the window’; sensory descriptions that emphasise vision; descriptions of ‘being in nature’; descriptions of ‘doing in nature’; barriers to sensory engagement; and meanings of being and doing in nature.

**Conclusions:**

Older people derive considerable pleasure and enjoyment from viewing nature, being and doing in nature which, in turn has a positive impact on their wellbeing and quality of life. Future research could usefully explore how sensory engagement with nature could be used to stimulate reminiscences of places and people, and evoke past sensory experiences to enrich everyday life and maintain a sense of self.

The protocol was registered with PROSPERO (CRD42015020736).

**Electronic supplementary material:**

The online version of this article (doi:10.1186/s12877-016-0288-0) contains supplementary material, which is available to authorized users.

## Background

Being in the outdoor environment experiencing nature, plants and wildlife are said to have various physical, mental and social benefits for older people [1, 2]. However, the outdoors can create a ‘dilemma’ [3] for older people in that it offers opportunities for physical activity and meeting people, but enjoying the many activities associated with the outdoors depends on the individual’s ‘embodied capacities’. These refer to “…what people can do with their bodies in relation to the social and physical environments in which they are situated” [4] (p.724). That older people do not spend enough time in the outdoors [5, 6] suggests that the outdoor environment can present barriers which, if combined with diminishing mobility and sensory acuity, can mean that older people have less capacity and less choice to be outdoors.

Since the older population is not homogeneous [1, 3] it seems likely that diverse groups within the older population will experience quite differently the ‘bodily opportunities’ and ‘bodily constraints’ [7] that the natural environment provides. Examples of different groups within the older population include those who live within the community and those who live in residential/nursing care; and older people who live with dementia, who can be still living independently with or without a formal diagnosis and those who need to have residential or specialist residential dementia care [1]. There are over 46 million people living with dementia worldwide and this is estimated to increase to 131.5 million by 2050 [8]. The majority of people with dementia are living in the community [9], yet outdoor space has rarely been conceived of as a ‘dementia setting’ [10] (p.361), with the result that people living with dementia can sometimes feel ‘out of place in outdoor space’ [11] (p.283). Despite the UK Government’s ambition that “everyone should have fair access to a good quality natural environment” [12] (p.44),

[t]he simple act of getting out into nature and experiencing the benefits this brings can often feel or be out of reach for many people living with dementia. As dementia progresses, many connections seem to be severed both with people and with places [13] (p.26).

So older people, and particularly those with dementia, may need support to enjoy the natural environment. Living well with dementia is a recent UK government priority [14] and arguably, being able to access and enjoy the outdoor natural environment should be part of what the ‘good life’ with dementia looks like [15].

There is a body of work on the outdoors and wellbeing but few studies have specifically focused on how the natural environment benefits older people and older people living with dementia. The existing evidence points to how older people benefit from the restorative effects of nature [16, 17] and to the beneficial health effects of the natural environment for older people living in nursing care [18], and for those living with dementia, with improvement in sleep patterns [19, 20] and in behaviour [13, 21]. Studies on therapeutic landscapes and mobilities have explored how places and moving from one place to another can contribute to wellbeing [22–24]. Gardens, domestic or allotment, healing or restorative, and care farms can be described as therapeutic environments and have been used for nature-based interventions with older people. Through ‘body work’ [23], such interventions can produce health and wellbeing benefits which are not simply about the benefits of physical exercise but also about what is sensed (heard, seen, smelt, touched) when the body actively engages with the natural environment [23].

The ‘sensory turn’ in the social sciences has led to a developing sociological literature on the senses and, whilst there has been some attention on sensory experiences in the outdoor natural environment [7, 25, 26], there has been little focus on older people’s experiences. There is emerging evidence that the natural environment contributes to stimulating the senses of older people with dementia [21, 27] and increasing recognition of the importance of holistic engagement of the senses in garden design, horticultural therapies and green care farms for people living with dementia [22, 28, 29]. Yet, “…little is really understood about the sensory experience for people living with dementia connecting to nature and how this may help to maintain their sense of self” [13] (p.3).

It is this link to sensory experience in the natural environment that this review aims to explore. The chosen approach was a systematic review of qualitative literature which was conducted to explore the extent to which such multi-sensory interaction is seen in research with older people, including those with dementia, about their interactions with the outdoors. Qualitative research was reviewed as it captures the holistic experience and meaning of engaging with nature for older people, and specifically seeks depictions of sensory experiences. We were particularly interested to explore if, and how, older people conceived of their contact with green/natural space in sensory terms and how this affected their experience.

This paper addresses the following questions:

- When using natural settings, how do older people describe their sensory engagement with the outside world?
  - ○ Are there different experiences for different groups of people (e.g. those with dementia)
  - ○ Are there ways in which these experiences can be enhanced?

## Methods

We conducted a systematic review and synthesis of relevant qualitative research. The protocol was registered with PROSPERO (CRD42015020736).

### Literature search

The search strategy was developed by an information specialist (SB) in discussion with the wider team. The strategy combined search terms for ‘older people’, ‘environment’ and ‘qualitative research’ using the AND Boolean operator and appropriate adjacency settings. Synonyms and Medical Subject Headings (MeSH) terms were identified by experimenting with different terms in the databases, with the aim of ensuring an appropriate balance of sensitivity and specificity. The identification of search terms was helped by considering the titles, abstracts and MeSH terms of key papers. The final search strategy was translated for use in other databases and limited to papers published between 1990 and September 2014, and written in English. The date limitation was to ensure that broad range of evidence was identified, whilst ensuring it was likely to be relevant to contemporary settings.

The following databases were searched: MEDLINE (Ovid), MEDLINE-in-Process (Ovid), PsycINFO (Ovid), CINAHL (EBSCO), GreenFILE (EBSCO), ProQuest Sociology, ASSIA (ProQuest), International Bibliography of the Social Sciences (ProQuest); HMIC (Ovid); Social Policy and Practice (Ovid). The database search results were exported to Endnote X7 and de-duplicated using the software and manual checking.

Two reviewers (NO, AW) screened the titles and abstracts to establish if they met predefined inclusion criteria:

- used recognised qualitative methods of data collection (e.g. interviews, focus groups) and analyses (e.g. thematic analysis, grounded theory);
- explored older adults’ (i.e. 60 years or over) sensory experiences with nature (which included wild environments such as moors as well as managed ones, such as gardens, parks and farms, and could include either directed or individualised engagement).

We used age 60 as a cut-off point because retirement is often seen as a time when people’s activity and behaviour may change, and we wanted to capture this for men and women in the period 1990 to date.

If they appeared to meet the criteria the full text of articles were obtained and further screened (by NO, AW). Forward and backward citation chasing was also undertaken. Resulting titles and abstracts were uploaded into Endnote where they were searched for three search terms: ‘qualitative’, ‘interview(s)’ or ‘finding(s)’ and then screened. The reference lists of review articles were scanned for further relevant articles based on their titles, as was the reference lists of all included studies. In addition, 20 relevant organisations were contacted by email (NO, AW) and asked to identify unpublished reports. These were charities and other organisations who focus on care and services for older people, including those with dementia.

Each included study was quality appraised using the Wallace criteria for qualitative studies [30] by two reviewers (NO, AW) and checked by the third reviewer (RG) (Additional file 1: Table S3).

### Data extraction

Each included article was read by two reviewers (NO, AW). One reviewer (NO) extracted information about the study aim, population and sample size, location and the type of intervention or programme. Details were then and checked by a second reviewer (AW). These details can be seen in Additional file 1: Table S1. Methodological details, which included approach to analysis, sample and type of sample and theory used, were extracted and recorded in Additional file 1: Table S2. The findings - themes and supporting quotations - were extracted from each included study and recorded in a table. This was read and discussed by three reviewers (RG, NO, AW).

### Data synthesis

Thematic analysis was used to synthesise across the qualitative studies, as they were descriptive in nature with little additional interpretation of findings [31]. Data in the form of quotations from older people (first order concepts) as well as author interpretations of data (second order concepts) were extracted. The articles and extracted data were read and re-read and the findings organised into themes. Details about the participants provided in the reports are reproduced here; first order (participant quotes) and second order (author interpretations) are also reported.

## Results

**Fig. 1:**
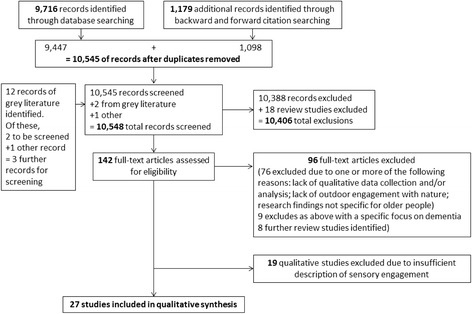
Screening Flowchart

The searches resulted in 10,548 study references, of which 142 were retrieved as full text (Fig. 1). A full screen text identified 27 studies that met the inclusion criteria; 19 further studies were not included in the synthesis because of insufficient sensory description although they did explore older people’s experiences in nature (see Fig. 1 for reasons for exclusion).

### Study characteristics

Eleven studies reported on programmes or interventions; three reported on garden projects, two of which were evaluations of therapeutic gardens for care residents with dementia and the third was a therapeutic garden walking intervention for older people experiencing depression. Three reported on horticultural therapy involving garden projects of varied duration but all using community garden settings. Six of the studies involved older people living with dementia, either in a residential setting or in the community, and two studies included both care home/nursing home residents living with dementia **and** those without. Eighteen studies reported on older people living in the community, one reported on older people living in their own homes **and** older people living in residential care. The studies involved over 1,081 older people (the total number is not clear) and the average age of the research participants was 72 years (though the mean age could only be calculated for 15 studies as the other studies gave insufficient detail) (See Additional file 1: Table S1).

### Study quality

All but one of the studies had a clear research question, most used appropriate study designs, and adequately described how the data were collected. In most studies the sample was drawn from the appropriate population and was adequate; and ethical approval and the gaining of informed consent from the sample were noted in most. Less than half of the studies noted a theoretical perspective and in those cases that did, it was difficult to tell if it influenced the study design. It was also difficult to appraise the data collection and analysis quality due to poor reporting in most studies (Additional file 1: Table S3).

### Qualitative synthesis

Two studies [32, 33] organised their thematic analysis around the categories of ‘being’ and ‘doing’ and this has informed the synthesis of the review findings. The thematic synthesis identified the following themes which are described in detail below:

- Descriptions from ‘the window’: appreciation of ‘green’ views from ‘inside’
- Sensory descriptions that emphasise vision
- Descriptions of ‘Being in Nature’:
  - ○ Being outdoors in the ‘fresh air’
  - ○ Being outdoors for social interaction and independence
  - ○ Being outdoors in peace and tranquillity
- Descriptions of ‘Doing in Nature’
- Barriers to sensory engagement with Nature
- Meanings of Being and Doing in Nature

Additional file 1: Table S4 shows which studies contribute to each theme.

### Descriptions from ‘the window’

Eight studies reported on the importance of viewing nature from ‘the window’ for both those living in residential/nursing care and those living in the community [24, 34–40]. For some care home residents, the more rural or green the views from the home, the better they said they would feel [34]. Views of greenery and the garden from bedrooms and communal areas were appreciated by residents and significant for their wellbeing [34, 38]. Similarly, older people living in urban neighbourhoods became familiar with their views of public green spaces, vegetation and plants and spoke of their favourite elements that they could see from their windows:

Woman (Age 80s, Coastal Town) – “So I’m quite fond of this shrub, tree, across the road here. I was speaking to the man yesterday and telling him once again how much I appreciated it. It’s across that way. It’s beautiful and it’s so well pruned” [35] (p.309).

As well as these ‘manmade’ green spaces, having views of open natural space with the possibility of seeing far away, rolling hills and mountains were greatly appreciated by residents and community dwellers, whether in urban or rural settings [35, 37, 38].

A seventy-nine-year-old woman who had lived in the same rural community all her life had beautiful views of open space and rolling hills out her window but the chair where she spent the majority of her day faced away from the large windows that look out over the front of her property. She had great difficulty getting up from the chair and connected to the world outside through a mirror she had set up on her coffee table. Through the mirror she could experience the outside world. She spoke of her connection to the natural world through bird watching [37] (p.165, author quote).

For older people living in rural environments, travelling by car increased their access to nature and they spoke of their enjoyment of seeing nature’s beauty [37]:

Mr Gardiner - “I love natural beauty. I always have. I feel close to nature. I never tire of it. When I’m driving, I like to look at what there is to see” [39] (p.140).

These excerpts show how for those older people, the opportunity to view the natural world through the window was their way of connecting with it. It is clear that green views had positive impacts on older people in terms of enjoyment and a sense of wellbeing. Although some authors have described this appreciation of nature and its ‘aesthetic beauty’ as ‘passive engagement’, it would seem that older people ‘actively’ seek to connect with nature despite their limitations [24, 37, 40].

### Sensory descriptions that emphasise vision

Twelve studies reported on older people’s visual descriptions of the outdoors [24, 35, 37, 38, 41–48]. This theme is close to the first theme, descriptions from ‘the window’, but has a greater emphasis on active engagement – ‘looking’ and ‘watching’ [24]. Whether indoors or outdoors, older people were reported as enjoying looking in detail at plants and flowers and being impressed by the beauty and intricacy of nature:

Women (Age 70s) Inner City Neighbourhood – “…And I think it brings you back to nature and makes you realise that there is more to life. Even just sitting *watching* flowers, looking at the flowers, the different colours, the different shapes of petals and all that, you could spend ages” [35] (p.309, reviewer edit, reviewer emphasis).

Similarly, observing and following the seasonal variations and changes in plant growth, flowering and colouring evoked feelings of wonder at “the marvel of nature” (participant quote) [24].

One interviewee in the Inner City Neighbourhood overlooked the park, and although she did not go into it much, she described in detail how she *watched* the changing seasons from her window, and the order in which different plants and flowers came into leaf and bloomed [35] (p.309) (author quote, reviewer emphasis).

These extracts suggest that older people’s greater focus on clarifying the detail of trees, plants and flowers was a compensation for being less active and having fewer opportunities to be outdoors.

Gardeners, in particular, spoke of their pleasure at witnessing new life and growth and the joy of planting and seeing new life emerge:

“I like to see…how they are growing so nicely. I was told that there was going to be large green peppers. I like to see things, like, what they are going to do?” [48] (p.97, edit in original).

In some cases, older people described how they connected to nature by watching wildlife and birds:

Woman – “I walk four to five miles every day…I can reflect and talk to myself…I am *totally absorbed watching* the birds and the wildlife and the different seasons”. [47] (p.389, edits in original, reviewer emphasis).

Elsewhere, walking was reported as a means of enhancing older people’s visual appreciation of nature and also as a way of keeping the mind alert:

“These walks have *opened my eyes* about the beauty that surrounds me and puts my problems and fears into a perspective that is easier to live with” [43] (p.257, reviewer emphasis).

“I go out about every day and *look around*. It keeps your mind working” [42] (p.343, reviewer emphasis).

However, there were cases where older people highlighted that their preference was to sit and watch nature through the window.

These extracts focus on how older people connect with nature visually and provides support for the idea that older people derive pleasure and joy from looking at and watching nature, whether they were outdoors walking in gardens or sitting observing nature from the indoors. That the older people were experiencing a ‘new’ or ‘fresh’ appreciation of nature and its lifecycle is suggested by the descriptions. It is possible that the choice to sit and observe nature was a reflection of physical ability but perhaps, it opened up the possibility for greater depth of absorption in the familiar and local.

### Descriptions of ‘being in nature’

#### Being outdoors in the ‘fresh air’

The benefit of being outdoors in the ‘fresh air’ was highlighted by many studies [32, 33, 35–38, 49, 50] and was closely associated with feelings of wellbeing. Breathing fresh air was believed to be good for both mind and body, and this was, in particular, highlighted by those living with early to moderate dementia [49]:

Participant – “It’s good, it’s good to have fresh air. I mean I clog my lungs up with pipe tobacco so I can get them *cleaned* out when I go outside” (p.195, reviewer emphasis).

Participant – “But er, yes I like to do that. I don’t think I can just sit all the time…it’s a change to get out you know. Well fresh air, makes you *think better*. If I’ve been walking around the garden, then I come back and I feel great” (p.195, edit in original, reviewer emphasis).

In some cases, residents reported that they wanted to ‘feel the fresh air’ or feel the weather - sunshine and wind [33, 36, 38, 50]:

Man – “It makes you happy, it’s a relief to get out and *feel that the weather is okay*, or if I feel like it I can take a walk – moving around is a *relief*” [33] (p.796, reviewer emphasis).

By being outdoors, the weather is much more than a visual perception and is experienced in a multisensory way; describing the weather as an experience of ‘feeling’ is perhaps because of the difficulty of disentangling how each of the different senses contribute to experiencing weather.

Being outdoors was perceived as a “…*fresher*, more airy and more beautiful” [38] (p.396, author quote and reviewer emphasis) experience than being indoors:

Resident – “Good…it *stimulates* you, brings you to life. When I go outside, I realise what a wonderful place the world is” [50] (p.e97, reviewer emphasis).

The freshness of the air was also perceived to be better and healthier in the countryside and at the coast [35]:

Participant – “…And I like the fresh air, the air seems to be better out in this area” [37] (p.163, reviewer edit).

The authors found that fresh air was regarded positively by older people enabling them to engage with, and enjoy, the natural world. ‘Fresh’ air made them feel alive by connecting them with the environment and making them feel part of the world. Simple pleasures such as feeling the sunshine and the wind by the sea were important ways of connecting to the outdoors [33, 38]. Arguably, being in fresh air is an inherently multi-sensory experience in that ‘air’ conveys feelings of wind or breeze, the sounds and smells of nature, and a sense of open space. However, this was not reflected upon in the studies; the benefits of being in the ‘fresh air’ were regarded as self-evident and were not explored by research participants or authors.

#### Being outdoors for social interaction and independence

The outdoors was appreciated by older people as it enabled them to participate in activities such as sitting, watching the surrounding environment, reading, drinking coffee, and having meals and picnics [33, 38, 40, 42, 45, 49–51]. There was a sense that being outdoors elevated ‘the everyday’ and also opened up opportunities to meet and talk to people:

Resident, Woman – “The garden is great because everybody can gather there. You get to know the people visiting other residents. Contact with other people is important” [38] (p.397).

For some nursing home residents, the social contact did not need to involve interacting with others: simply being outdoors and observing others was perceived as beneficial [33, 38].

The importance of gardens allowing for both social interaction **and** privacy was observed in an evaluation of a secure garden for people with Alzheimer’s Disease:

“Although many structured activities were observed in the garden, we observed that it was when clients were independently using the garden that they tended to interact with one another which may have enhanced the pleasure of their experience in the garden” [51] (p.426, author quote).

Enjoying being outdoors was in some cases linked to appreciating the contrast of being outdoors away from the restrictions of being inside the residential home. In one study residents expressed a preference for being outdoors liking the “…the sensation of being free and out in the open” [50] (p.e96, author quote):

Woman - “I’d feel like a prisoner if I wasn’t able to go out …it would be horrible, it would be terrible, I don’t want to be trapped inside, never” [33] (p.796, edit in original).

The sense of freedom from being outdoors was also described by older people in a study of those in the early and moderate stages of dementia:

“Fresh air, exercise, meeting people – I usually find someone to talk to en route and *escaping* yes yes…it’s just nice for a change of scenery yeah” [49] (p.195, edit in original, reviewer emphasis).

In one study, older people living with dementia had participated in woodland walks, and they described how it was important for them to connect with the wider world by being outdoors [45]:

“It is important to get out into the larger world and community so you don’t get shut away” (p.12).

“Got to come out into the world to see what is going on” (p.12).

Being outdoors, whether alone or with others engaged in activities, was regarded by older people as beneficial and appeared to contribute to maintaining their quality of life. The opportunity to be outdoors and doing activities outdoors was important for maintaining connections to the rest of society and self-confidence, particularly for those living with dementia [33].

#### Being outdoors in peace and tranquillity

Being outdoors was described in nine studies as enjoyable because of the peace and tranquillity experienced [24, 32, 35, 37, 42, 43, 46, 48, 52]. Tranquillity seemed to refer not only to a place that was quiet but also to a state of mind that was peaceful or undisturbed. All of these studies focused on gardens, both domestic and allotment, and included garden projects/programs. The domestic garden was seen as an important private space where older people could experience the peace and tranquillity of nature [24]. Similarly, allotment gardens were perceived as peaceful, offering relaxation and freedom from stress [32].

The peace and tranquillity to be found in gardens that were located in urban settings was reflected well in an article that reported on an evaluation of a community gardening program [48]:

Participant – “It is so relaxing. It just makes me feel peaceful inside. I love doing it. It’s second nature to me. I really love, love the land, love the earth, I just love…I grew up on a farm, first of all. There’s nothing like it. Sometimes I just go out under the tree and I sit there. I appreciate the trees and the grass. Everything so much. It’s kind of hard to explain, but it do. It relaxes your mind. It gives you that calmness, you know? You can’t always get that everywhere, especially in the city. But try going to the park, down by the water, or gardening” [48] (p.95, edit in original).

It was clear from these studies that simply ‘being in the garden’ experiencing nature was as important as engaging with nature during gardening activity in inducing feelings of calm [24, 32]. Walking in the garden was also shown to have therapeutic effects for older adults experiencing depression. Spending time alone for the sole purpose of relaxation and reflection in garden settings generated a sense of wonderment at the beauty of nature and a feeling of peace:

Participant – “The overwhelming beauty and peacefulness of the garden gave me a new approach to old troubles. I hope this will carry me forward in my life. The variety of water, flowers, trees, sky and sun were exhilarating. The lakes had a tranquil effect and the waterfalls gave me a sense of cleansing” [43] (p.257).

These highlight how garden walking can be a way of mind and body connecting with nature and it seemed to increase the intensity of the walker’s engagement with nature. Although these excerpts do not specifically mention pleasant sounds, implicit within the descriptions is the absence of unwanted sound or noise that would inhibit feelings of calmness and relaxation. It seems possible that the more the participants connected to the peaceful and tranquil environment, the greater their feelings of tranquillity and calmness, and appreciation of the natural environment.

Although quiet outdoor spaces were clearly appreciated and in one study, older people suggested that quiet became more important with age [35], it was not always the case that noise was perceived as negative

“…some individuals, especially those living alone, stressed that it was possible to be too quiet, and that they liked to be aware of some life around them such as people passing by and children playing” [35] (p.305, author quote, reviewer edit).

and where it was in harmony with the environment (e.g. birdsong in the garden) it was sought out:

Woman (Age 93) – “I sit out there and there is an olive tree in the garden and I prefer to sit outside, it’s not so lonely being outside in the open. You can hear the birds, not so lonely as always being by yourself inside” [52] (p.1161).

These descriptions provide support for the notion of the garden as a therapeutic environment and arguably, the feelings of calmness and relaxation capture, albeit implicitly, the harmonious multi-sensorial experience of being and moving in the garden free from stress.

### Descriptions of ‘doing in the outdoors’

Seven studies reported on older people’s particular engagement with nature through gardening activities [24, 32, 36, 37, 42, 46, 48, 53]. For those older people living in their own homes, the garden was the often the ‘primary site’ [36] (author quote) from which they could engage with nature.

Woman (Age 78) – “I wouldn’t want to live anywhere else…I like the things there are to do here. I enjoy them…I like, well I’m always gardening. I don’t have a vegetable garden now, but I did up until last year. I love gardening. I love the freedom of it, being able to go and walk and go in the field and things like that” [37] (p.163, edit in original).

Descriptions of ‘doing’ gardening encompassed a number of physical tasks such as digging, raking, planting, watering, bending, pruning, chopping and harvesting [24, 32]. Due to the range and intensity of body movements involved in performing these tasks, gardening was regarded as an enjoyable, all-round form of exercise, which often helped lift a bad mood:

“It *keeps me on, my feet and moving*…it’s the only place you can get your hands dirty and your heart clean” [53] (p.50, edit in original, reviewer emphasis).

Margaret - “Pruning things is brilliant, there’s nothing better than just chopping things down, it’s brilliant!…You feel better afterwards than beforehand…sometimes it is an effort to go to the allotment, but I just feel so much better when I’ve been, better mood” [32] (p.117).

Planting crops and watching them grow were reported as rewarding activities:

William – “I like growing things, I like creating things, anything from just buying a packet of seeds and putting them in a seed tray or something and one day you have a look and there’s lots of little green spikes” [32] (p.120).

“I garden for the sheer pleasure of it…taking and planting cuttings and giving them a chance at new life” [46] (p.12, edit in original).

Harvesting and eating the fruit and vegetables grown were also highlighted as the very tangible benefits of gardening [42, 46, 48, 53]:

“The good thing you get out of it once you have grown it, tended to it, you can enjoy eating it” [48] (p.95).

Yet for some, it was not just about having the end product that tasted different but was also about connecting with a living, growing thing

“Because you, your food tastes different your tomatoes taste different, there’s no pesticide on them. Everything you plant you know you’ve grown it with *your own hands* and you watch it grow, that’s one thing, you go out some days it’s light green the next it’s red, all the little beans and soon you have a full grown garden. It doesn’t have to be perfect. It doesn’t have to be perfect, *it’s just you’re a part of that*” [48] (p.95-6, reviewer edits).

and appreciating the earth and soil:

“I used to sit in the dirt. Just sit in the dirt. And do my gardening. Because it’s me. Barefoot, sitting in the dirt, enjoying the earth. And it has a good feeling…the soil. It smells good…Did you ever smell the soil? Especially after the rain? It smells so good. And don’t look at me like I’m crazy” [48] (p.97).

These extracts highlight that through ‘doing’ gardening, older people appreciate that the natural environment is integral to what they do. This is reflected in their descriptions of working in tandem with the cycles of nature – growing, nurturing, and harvesting - and asserting control over nature by removing what is not wanted. Their gardening experience involving the performance of physical tasks was embodied and multisensory.

In one study, outdoor adventure (activities ranged from half day walks and bike rides to multi-day pack-carrying trips and biking expeditions) offered older people “…intense, embodied experiences in natural places” [54] (p.662, author quote) as demonstrated in the following quote:

Woman (Age 68) – “I love the mud – getting muddy…It is the awe, the smells, the earth, the lovely mossy smell and the ferns and the bush, the sunset, the sparkle of the sea and those things – the wow factor…I like the battle with the elements, wind, rain, clouds; I don’t mind getting wet or walking in the rain” (p.657, reviewer edit).

The description offered by this participant shows that an older person with greater physical ability or ‘embodied capacity’ can experience heightened sensory awareness in the outdoors and in this case, it is almost a ‘celebration’ of being in the natural environment, even when ‘getting muddy’ or ‘getting wet’.

For some, the natural environment was also a motivator to walk [24, 35, 43, 47] and had therapeutic benefits:

Woman – “…It refreshes me…I walk and walk if I start to feel sad and lonely…my husband died seven years ago” [47] (p.389).

Ted (Age 69) – “Most mornings I take the dog for a walk out. There’s a clique of us, we meet up by the river…I go for a walk, maybe three or four miles – it’s a leisurely walk, you know? And we put the world to rights – it’s *a good stress reliever* I would say” [24] (p.1785, reviewer edit).

Rather than these descriptions noting the sensory experience of walking, the focus is on the benefits of walking, whether alone or in the company of others (or pets), to wellbeing and particularly, mental wellbeing. Walking afforded opportunities for social encounters on the move and the enacting of routines which were perceived as having a positive impact on quality of life.

### Barriers to sensory engagement with nature

Whether living independently or living in a residential/nursing care setting, older people can experience challenges to engaging with the outdoors and the natural environment. These included sensory impairments such as deafness and blindness and poor physical health leading to difficulties with walking and reduced mobility [33, 36, 50].

The weather could be a determining factor as to whether older people went outdoors and winter weather was associated with slipperiness resulting in a fear of falling which prevented them from going outdoors [33, 34, 38]. Whilst for some, sunshine and warmth were an ‘attraction’ and ‘precondition’ for going outdoors [38] (author quotes), others expressed fears of sunburn and heat discomfort. Such differences may also be seasonal in temperate countries. Accessing nature for some older people was not always positive because ‘microclimatic factors’ [51] (p.426) such as glare, draughts, availability of shade and temperature extremes could mean that they experienced considerable discomfort in the outdoors [36, 38, 51].

Viewing nature from indoors could also be a challenge for older people:

“Building configuration also influenced access to nature…The orientation of rooms within a building and their subsequent use may influence interaction (e.g. whether a window faces onto a garden or the street.) Similarly, configuration of furniture may restrict viewing possibilities” [36] (p.64, author quote, reviewer edit).

The importance of viewing and watching nature for older people means that the quality and variety of views are key to provoking interest and can also influence the decision to go outdoors [55].

Two studies reported that older people simply had no interest in being outdoors or lacked inclination or initiative [36, 50]:

“If I had my choice, I’d stay inside as much as I can, sit in my chair and doze” [50] (p.e97).

“Don’t need to go outdoors, I like to read and write” [50] (p.e97).

It was apparent that lack of initiative and concentration was also an issue in accessing nature for people living with dementia [36].

How dementia impacted on older people living in the community and their ability to be outdoors was reported in several studies [33, 36, 49]. Specific symptoms associated with dementia such as deteriorating memory, confusion and disorientation [49] led to feelings of panic, loss of confidence and anxiety which all contributed to decreasing outdoor activity. Deteriorating memory could encompass forgetting about going outdoors, forgetting about having been outdoors, forgetting how to do something (e.g. losing tools/equipment needed to do gardening) and forgetting how to return home [49]:

Man – “I can’t find it…you think you might find your way, but the fear of not being able to becomes a mental block” [33] (p.797, edit in original).

The problem of disorientation experienced by older people with dementia affected their ability to be outdoors:

Woman – “When you go outside and you’re out there, it’s like walking in your sleep” [33] (p.797).

Opportunities to access to the outdoors for older people living at home and in residential/nursing homes were often dependent on the availability and willingness of carers to assist them. The demands placed upon care workers’ time and resources could be a significant barrier for older people to being outdoors [36, 50].

In one study, staff attitudes towards older residents’ use of outdoor natural spaces such as gardens were shown to be a barrier [51]. Staff who lacked ‘green fingers’ or experience of gardening found it challenging to generate gardening programmes for residents:

“The creativity involved in planning and implementing garden-based programs added to the complexity and challenge of their work…the time involved in doing innovative creative gardening programming was sometimes difficult” [51] (p.425, author quote, reviewer edit).

Being ‘comfortable’ and ‘feeling at home’ [38] in the outdoor environment emerged as important for care home residents but, as highlighted in one study, ‘the lack of privacy and autonomy’ [50] (p.e97, author quote) acted as a significant deterrent to being outdoors. Independence could be further curbed by external doors to gardens being kept closed or locked in order to prevent residents going out by themselves, which could then be problematic for other residents:

Woman - “I don’t like it when the door is locked because sometimes I have to wait for a long time before someone can help me get out” [38] (p.397).

Poor maintenance of gardens and other outdoor spaces were also identified as major barriers to use [38, 51]; uneven ground cover could lead to tripping hazards and made the use of walkers difficult, thereby increasing the risks of falls.

Older people still living in their own homes were more likely to be able to influence the layout and design of their gardens to accommodate their decreasing capacity to garden [36]. Two studies reported on how awareness of their declining physical abilities meant that older people made preparation for old age by adapting their gardens so they could still use and work in them despite being less physically able:

Ralph (Age 72) – “I’ve got it (the garden) slabbed and it’s in squares right the way round. I’ve done this about four years ago, when I knew this (old age) was coming on and I said ‘well I’m going to get it done and make it my ‘half hour garden’. I’ve got my greenhouse and I’ve got plants in it coming through and then they’re just to plant out. Once they’re planted out, the garden bit’s done. That’s the way I look at it like” [56] (p.1787, edits in the original).

Woman (Age 78) – “Now I have only a very small garden but have lots of window boxes, large pots and troughs which I look after…specially the troughs at the kitchen window which brighten the day. I also have lots of plants indoors which I get lots of pleasure from. A house without plants is empty in many ways I think. I enjoy the garden without any hard work on my part, which is great [44] (p.330, edit in original).

Older people made other modifications in order to continue gardening such as doing shorter but more frequent gardening sessions and carefully planning how they could accomplish tasks either by using modified tools or seeking assistance with tasks that they could no longer do on their own [46].

So those living in their own homes, in contrast to those living in residential care, had more control of their environs and were able to make adaptations that suited their needs to facilitate constrained use of outside spaces. For those who could no longer continue to do gardening, the garden could still offer an outdoor space for ‘pottering’:

Woman (Age 81) – “I am now 81 and arthritic, but I can still potter or just sit under the chestnut tree with sewing or a book and but again may be just dozing” [44] (p.332).

Older people’s relationship with the natural environment could be altered by relocation [56] which could be positive when managing the garden was a challenge and negative when access to green space was lost:

Ada Jennings (pre-move) – “I’ll remember the nice space, the nice garden we had. But I won’t really miss the garden because it was too much for me” (p.383).

Bob Flynn (post-move) - “I loved the woods down there. That’s what I miss more than anything…watching the birds, and the squirrels, and all that, and all the animals down there” (p.385).

Relocating from a rural location to town could facilitate easier access to services but could lead to the ‘loss of aesthetic capital’ [37] (p.166, author quote), in terms of closeness to natural beauty. One study reported on how some older people had relocated from the country to town and had a longing for nature [37]:

Woman (Age 85) - “I do miss the country. I always loved being in the country and seeing all of the birds…the various variety of birds. We had bird feeders all over the place and raccoons that live under the porch. And they’d come to see us…We really felt we were a part of nature I think” (p.166, edit in original).

### Meanings of being and doing in nature

Nine studies discussed the meanings of being and doing in nature [38, 39, 42, 43, 46, 47, 53, 57, 58]. In two of them, older people described how viewing nature and being in nature gave them a feeling of a higher power and a feeling of being part of something much larger; for some explicitly spiritual or religious [47]:

Woman – “When you look at the sunset, you really know that somebody’s taking care of us…I just feel that I can get up and start something” (p.389, edit in original)

and for others, oneness to nature [43]:

“I feel as though my life has meaning now, I am part of something much larger-nature, the grandness of being alive, it is exciting and I see it as a new beginning” (p.257).

Spirituality and the relationship with the earth and nature were also highlighted by the participants in an evaluation of a gardening project [53]. Gardening was described as:

“the most worthwhile thing you can do. It teaches you how to grow. Like the parable of the seed, it teaches life cycles” (p.50).

Being in nature had the power to evoke memories and take older people to different times and places:

Mr Gardiner (Age 90) - “I often think of my past…My early life was quiet, simple and orderly and I lived with nature…” And, “I loved that quiet rural life. We liked to be in that pastoral atmosphere. We collected birds’ eggs. We hunted. We swam in a pool and wandered in meadows. We felt free to roam anywhere. We were known” [39] (p.140, edit in original).

For older people who were living in residential/nursing care, being in natural surroundings that connected them to their past was important for giving ‘a feeling of being at home’ [38] (p.397, author quote).

“The possibility to connect to the past through the surroundings varied from person to person…this was sometimes a matter of recognizing the scent of a flower” [38] (p.397, author quote, reviewer edit).

In one study that feeling of ‘being at home’ was created for care home residents with dementia by integrating familiar objects from their home into the design of a therapeutic garden:

“I like it all. The fountain, the fish, the memory boxes – everything. The table and chairs in the sunroom came from my lounge room at home, you know. We all sit around it and talk” [57] (p.506, edit in original).

Linking back to childhood experiences of nature perhaps gave a sense of continuity which was also important for connecting with the next generation by ‘handing down’ or sharing ‘nature knowledge’.

“One of my grandchildren…she’s into flowers. It makes me kind of feel like she’s part of…well, coming from my branch of the family” [53] (p.49, edit in original).

This was also highlighted in a study of Secwepemc Elders in British Columbia, Canada who sought to share their cultural tradition of growing their own food with children:

“I had some Elders visit the greenhouse that we built and they brought a bunch of little kids, and well the little kids they never saw tomatoes like that, hanging from the roof, eh? Twelve feet high. And then we went to the garden and we dug potatoes and one little boy said, ‘What is that doing down there?’ He thought potatoes just – these are little kids, like we’re growers, originally our people are growers. And the kids couldn’t believe this, so they’re in there digging the potatoes, running all over the garden, they had more fun then – but you know what really hit me, they thought potatoes come from Safeway, they thought tomatoes come from Safeway [general laughter]” [58] (p.102).

These excerpts highlight the significance of being connected with nature and how this could in turn facilitate connections with the past and the future (younger generations), and with cycles of nature and cycles of life. They also show that older people found opportunities “…to reflect on life’s larger questions such as one’s priorities, goals, and one’s place in the overall scheme of things” [59] (p.166), suggesting the restorative potential of the natural environment [47].

## Discussion

This systematic review explores the qualitative evidence on the sensory engagement of older people with the outdoors and natural environment. The findings identified six themes around sensory engagement with the outdoors and natural environment. Older people’s sensory descriptions centred on the visual dimension of their sensory engagement and their multisensory experiences of ‘being’ and ‘doing’ in the natural environment. The significance of the visual dimension in older people’s descriptions of their sensory engagement with nature was that, whether through physical limitation or through choice, they could absorb the visual appearance of their natural outdoor environment from indoors, at all times and in all seasons. For those who experienced difficulty accessing the outdoors, including those in nursing homes, viewing nature such as hills, mountains and trees from the window could provide “…long-term contact with the natural environment” [60] (p.540). Care home staff could facilitate such contact by ensuring that residents are able to maximise views of gardens and other outdoor spaces, and by encouraging planting and wildlife into these spaces.

Older people’s descriptions of ‘being’ outdoors in nature were both explicitly multi-sensorial, demonstrating the intermingling of the senses such as touch and smell, and also implicitly multi-sensorial. For example, rather than the descriptions of being outdoors in the ‘fresh air’ explicitly highlighting the different sensory dimensions, they tended to describe it as a ‘feeling’. This may simply reflect that fresh air is rarely something we contemplate [61] but the sensation of air is multi-sensory:

listening to the outside and smelling it…is not just an auditory and emotional practice…It is motoric as well, *kinaesthetic*. The perception of air, such as listening to sounds in the air, its smells, or sensing the wind and raindrops on the skin may be compared to the perception of music [61] (p.184).

Similarly, the descriptions of being outdoors in ‘peace and tranquillity’ did not explicitly detail sensory perceptions but arguably, implicit within the description of tranquillity is the notion of a harmonious environment “…with no single sensory perception dominant” [62] (p.1032). This reinforces not only that the senses are intertwined with each other but also the need to understand “…how the senses interact with each other in different combinations and hierarchies” [63] (p.1).

Older people’s descriptions for ‘doing’ in nature, reflected the embodied nature of the activities undertaken, whether it was gardening or outdoor adventure. Gardening involves ‘doing’ with the hands (and using tools), touching and feeling plants and the soil. Encountering ‘the earth’ – soil and mud – seemed to generate a ‘heightened sensory awareness’ [25], particularly in terms of touch and smell and also, in some cases contributed to a feeling of ‘oneness with nature’.

Whether it was viewing nature, being or doing in nature, descriptions offered by older people stressed the pleasure and enjoyment they had from connecting with the natural environment. Pleasure and enjoyment have been identified by older people as one of the underlying attributes that gives their lives quality [64] and pleasure has also been acknowledged as “…a salient but under-researched indicator of quality of life in nursing home residents with dementia” [65] (p.403). It has been argued that for those older people living with dementia, the potential for happiness lies in embracing the ‘experiencing self’ [66] and having ‘momentary pleasures’ in the here and now [15]. Furthermore, it has been suggested that for older people living with dementia in nursing care, the experience of pleasurable moments is a skill that should be continually practised so that people maintain the ability to experience pleasure [65]. The sensory descriptions of being in nature suggest that older people appreciated the ‘uncomplicated aspect’ of being in nature and supports the notion that the natural environment can play a significant role in facilitating pleasure and enjoyment which may also be experienced as restorative. Simply being in the natural environment may bring pleasure in that it requires little effort, or ‘effortless attention’ [67], which is important for older people living with dementia who are affected by a decline in attention [28, 68].^.^ In order to facilitate these benefits and to fulfil the UK Government’s ambition of enabling access to good quality natural environments, care needs to be taken to ensure local green spaces are accessible to a range of older people, including those with mobility or other difficulties.

Despite the variation in older people’s embodied experiences of the outdoors, the sensory experiences were important for making them feel connected, being part of ‘ordinary life’ and even belonging to the wider world. The desire to feel connected to nature and the outdoors seemed to be as important for those living with dementia in the community as for other older people. This is in keeping with other research on meaningful activities for people with dementia [69, 70]. A feeling of connectedness, that is, “the perception of a positive or harmonious linkage between one’s sense of self and one’s experiences of relationships, agency, wellness, and place” [71] (p.28) was found to positively influence quality of life for people living with dementia in a recent review study.

Given that the review highlights older people’s desire to connect with the outdoors and enjoy sensory experiences and that these appear to have positive impacts on mood, quality of life and wellbeing, it is reasonable to conclude that older people should have the opportunity to engage in whatever experience is desirable and appropriate. From the evidence collected in this review, it is clear that those older people living in the community were more in control of their environs than those older people living in nursing/residential care and could adapt both their practices and environment to enable access to the outdoors. Specifically within residential/nursing care, staff commitment and support is essential for enabling residents to engage with the natural environment. Older people appreciated being in nature for the sense of freedom and independence but residents could find that limited staff resources and attitudes curbed their access. Residents in care need the practical support of others to participate in everyday activities and in caring for older people, the concept of ‘assisted autonomy’ [72] opens up the possibility for recognising and enabling older people to exercise agency in their lives. The need for a better understanding of the reasons for limiting garden accessibility in the residential/nursing home setting has already been identified [21] and is essential if older residents’ sensory experiences are to be enhanced.

The evidence also suggests that older people need to feel comfortable in the outdoor environment; a pertinent issue for residents relates to garden design (and maintenance) with the need for a ‘variety of microclimatic areas’ within gardens enabling residents to be in different parts of the garden according to whether they need sun or shade. The literature is awash with recommendations for garden design in the residential/nursing care settings, but it would seem that there is still a considerable lag between recommendations and practical implementation. One design possibility for care settings is to experiment with ‘transition zones’ or ‘place making at the building’s edge’ [73] in order to reduce “…the physical and psychological transition between indoors and outdoors” [55] (p.41). This could mean extending rooms into the garden by constructing patios, conservatories and protected balconies [74] and by providing places to sit or stand while looking to the outdoors, the transition between indoors and outdoors is mediated for residents.

That the descriptions of older people’s sensory experiences often did not explicitly discuss the senses is not surprising as difficulties in talking about sensory experiences have been well documented [25, 26, 75]. As already noted, 19 accounts of older people and the outdoors were excluded from the review because this element was not discussed at all. The significance of the visual dimension of older people’s sensory engagement may reflect that vision is the privileged sense in contemporary culture [26, 76] and therefore, the vocabulary for talking about the visual in nature is readily available. However, this should not detract from accepting, as described in the first two themes, that older people can derive “….both instant and longer lasting pleasure” [26] (p.326) from viewing and looking at nature. This is supported by other research that suggests that ‘nature in the window view’ played a significant role in residents’ wellbeing [60].

## Review limitations

What is experienced through the senses cannot be communicated easily to others for at least two reasons: the senses are part of people’s everyday lives and may seem too mundane to describe, and often sensory experiences may be fleeting, which can mean that they slip away from conscious reflection and are difficult to articulate [77, 78]. The methodological challenges this presents for researchers and the possibilities of combining traditional qualitative methods with more creative approaches like visual and sensory ethnographies, diary techniques and mobile methods such as ‘go-along’ interviews have been receiving increasing scholarly attention [79–83]. Accessing the sensory experiences of those living with dementia by interview is clearly a challenge for researchers and in a number of studies often carers or family members were interviewed. Yet, it is possible for people “…even with fairly severe dementia to express themselves meaningfully in words; very careful listening is necessary, with close attention to metaphor, oblique allusion, and the combination of the verbal and non-verbal registers” [84] (p.15). Most of the studies in the review used standard qualitative methods but the evidence examined in this review generally lacked depth, particularly in how the data were reported and analysed. A number of the studies were mixed methods studies, combining quantitative and qualitative methods, and the qualitative part of these studies did not, in many cases, yield rich, descriptive data. The analyses of the qualitative data did not always result in first order quotations and in a number of studies the qualitative data were presented numerically with frequencies and graphs – ‘quasi-statistics’ – which is controversial in qualitative research [85]. Much of the data on the appreciation of engaging with nature (e.g. being outdoors in the fresh air) seemed to remain unexamined and accepted as self-evident by the authors and not subjected to deeper analysis. The analysis of the sensory data could have been enhanced by drawing on appropriate theories such as phenomenology and embodiment. There is a need for high quality qualitative research to understand older people’s sensory engagement with the natural environment.

Although we used a comprehensive search strategy, identifying over 10,000 references, it is possible that we missed relevant studies, given that the research is spread across a number of disciplines, the language used to describe and index these types of papers is varied and the topic area is likely to employ diverse descriptions. We have attempted to mitigate against this by using additional methods, such as citation chasing and contacting relevant organisations.

Quality appraisal for systematic reviews remains a contentious issue. Whilst most agree that some way of distinguishing high quality work from poor is appropriate, there remains a lack of consensus about which criteria are most important, and these differ between disciplines, epistemologies and theoretical frameworks [86]. This means there is also no agreed set of reporting standards for qualitative research. Limited word counts for many journals often lead to poor reporting, but it is unclear whether this is indicative of poor study conduct. This review found that studies frequently failed to supply full details about how data were collected and analysed. Nevertheless, our synthesis identified a number of common themes all of which occurred in a number of the included studies, and all of these were supported by at least one study that scored highly on our quality appraisal tool. This increases our confidence in the validity of our synthesised findings.

## Conclusion

This synthesis demonstrates that older people derive considerable pleasure and enjoyment from viewing, being and doing in nature which, in turn has a positive impact on their wellbeing and quality of life. Although sensory loss is prevalent in older adults [87] and can clearly prevent them from participating in activities they enjoy, the findings from this review suggest that older people can adapt how they engage with nature and continue to have pleasure and enjoyment from their sensory experiences. Despite the increased scholarly interest in the senses and sensory experiences, the topic of older people’s sensory engagement with nature is currently under researched. The senses and bodily experiences are now being recognised as central to the experience of living with dementia [88], opening up “…new possibilities for interpreting and understanding dementia” [89] (p.283). Future research could usefully explore how sensory engagement with nature could be used to stimulate reminiscences of places and people, and evoke past sensory experiences to enrich everyday life and maintain a sense of self.

## Additional file

Additional file 1: Supplementary Information. (DOCX 39 kb)
