# Supplementary material for: How do older people describe their sensory experiences of the natural world? A systematic review of the qualitative evidence
Source: BMC Geriatr. 2016 Jun 1;16:116. doi: 10.1186/s12877-016-0288-0 (PMC4888483; doi:10.1186/s12877-016-0288-0)
Supplement: Additional file 1: — Supplementary Information. (DOCX 39 kb) [file 12877_2016_288_MOESM1_ESM.docx]

**Additional file 1**

**Table S1: Details of included studies**

| **Author(s)** | **Aim** | **Population and sample size** | **Location** | **Programme**  **/Intervention** |
| --- | --- | --- | --- | --- |
| Alves et al (2006) [41] | To examine the cultural differences in nature-related activities among Hispanic and Anglo-American elderly. To analyse the content of nature-related activities based on selected attributes of quality of life. To provide culturally sensitive recommendations for future-related health interventions in institutional settings. | 15 Spanish-speaking Hispanic and 15 English-speaking Anglo-American elderly people (institutionalised) | USA | None |
| Bengtsson & Carlsson (2013) [39] | To describe older persons’ experiences of outdoor environments at nursing homes in terms of what factors are important and in what way they are important. | 12 residents from three nursing homes (74-96 years; M = 86 years). No details on gender. | Sweden | None |
| Bhatti (2006) [44] | To highlight the importance of garden and gardening to older people. To understand the importance of ageing on the social construction of home, in which the garden is a significant site. | Mass Observation Data Archive – Garden and Gardening Directive (244 returns) & Growing Older Directive (429 returns) (15 extracts used) | UK | None |
| Boyes (2013) [53] | To explore the ways older people engage in outdoor adventure activities in a community-based programme | 6 older adults (63-80 years) (4 women & 2 men) | New Zealand | Third Age Adventure Programme ( had been running for 5 years) |
| Burton & Sheehan (2010) [34] | To identify the aspects of care-home design that appear to have the greatest influence on the wellbeing of older residents. | 81 residents in 20 care homes (M = 85 years; 59 women, 22 men) | England | None |
| Butler & Cohen (2010) [38] | To examine how elders living in nonmetropolitan regions in northeastern United States perceived their rural surroundings and the contribution of nature in their overall wellbeing. | Study 1 – 66 participants (34 volunteers and 32 of their clients; 62-99 years, M = 78 years; 54 women, 16 men); Study 2 – 8 participants (70-95 years; 5 women and 3 men) | USA | Programme – study 1 was a federally funded elder-helping – elder volunteer program, the Senior Companion Program. |
| Day (2008) [35] | To explore the ways in which the local outdoor physical environment may support or challenge older people’s health. | 45 older people (62-over 90 years). No details on gender. | Scotland | None |
| Duggan et al (2008) [49] | To report on the voices of people with early to moderate dementia living in their own home concerning their use of the outdoor environment and how dementia impacts on it. | 22 people with early to moderate dementia (71-84 years) (11 men & 11 women) | UK | None |
| Durvasula et al (2010) [50] | To ascertain the current and early life attitudes to sunlight exposure in older people living in intermediate care facilities. To determine the participants’ knowledge of vitamin D and its effects. | 57 residents (70-107 years) (39 women & 18 men; M = 85 years) | Australia | None |
| Edwards et al (2012) [87] | To evaluate whether a therapeutic garden can improve the quality of life of aged care residents with dementia. | 10 residents – 4 had severe dementia, 3 had moderate dementia, 3 had mild dementia (9 women & 1 man) (79-90 years) unclear how many residents themselves were interviewed | Australia | Project – therapeutic, interactive, sensory, wander garden created to increase quality of life for residents |
| Gibson et al (2007) [36] | To determine if and how nature was important to people with dementia. | 26 people with dementia: 16 with mild to severe dementia living at home; 10 living in residential homes (9 women & 7 men) (4 living alone, 12 with informal carer). | England | Programme – INDEPENDENT Project, EPSRC funded EQUAL 4 consortium project which investigated enabling environments for people with dementia. |
| Hassink et al (2010) [37] | To find out which characteristics of care farms are important for different client groups and whether care farms can be good examples of empowerment-oriented and strength-based practices for different client groups leading to an improved quality of life. | 12 frail elderly people (no details on gender) | Netherlands | None |
| Hawkins et al (2013) [32] | To investigate the benefits to health and wellbeing of allotment gardening in a community-dwelling older adult sample. | 14 allotment gardeners (54-82 years; M = 65.8 years) (10 men & 4 women) | Wales, UK | None |
| Heliker et al (2001) [54] | To demonstrate the feasibility and effectiveness of horticulture therapy and the perceived meaning and outcome on wellbeing of a structured gardening intervention on two groups of elders in two culturally diverse settings. | 24 volunteers/community dwelling elders (63-90 years): Group A comprised 12 volunteers aged between 67-90 years; M = 79 years (7 women & 5 men, 10 were African-American and 2 were Caucasian); Group B comprised 12 volunteers aged between 63-83, M = 70.1 years (11 women& 1 man, 9 were Caucasian, 1 as African-American and Caucasian and 1 was Hispanic). | USA | Intervention – three month gardening project comprising 12 classes |
| Hulko (2014) [88] | Two qualitative research studies undertaken with more than 33 Secwepemc elders in the Interior of British Columbia, Canada: i) to determine Elders’ views on memory loss and memory care in later life; ii) to build nursing capacity to care for Elders in a culturally safe way through an educational intervention involving storytelling that was developed in collaboration with Elders. | 33 Secwepemc elders in the Interior of British Columbia | Canada | Educational intervention involving storytelling that was developed in collaboration with Elders. |
| Jansen & von Sadovszky (2004) [47] | To identify the restorative activities of community-dwelling elders | 30 community-dwelling elders (65-92 years; M= 74.8) (28 women & 2 men) | USA | None |
| Lovering et al (2002) [51] | To gain a better understanding of how specifically designed outdoors spaces (developed to improve the quality of life of people with dementia) are used and the objectives of their design fulfilled | 100 people attending a day programme (39-96 years, most over 70 years) (not clear how many were observed during research) | Canada | Garden was part of a day program for community dwelling seniors with Alzheimer’s Disease. It operated 24 hours per day, 7 days per week. |
| McCaffrey et al (2010) [43] | To determine the effect of garden walking and reflective journaling on adults who were 65 years and older with depression. | 40 adults (M =71.3 years) | USA | Garden walking intervention |
| Mapes (2012) [45] | To share the key findings of the Wandering in the Woods action research project | 24 (living with dementia in care settings) | England | Pilot Project to enable three groups of care home residents living with dementia to get out of the care home and to re-connect with nature. Designed and delivered collaboratively between the Woodland Trust, Dementia Adventure CIC and Caring Homes. |
| Milligan et al (2004) [24] | To investigate benefits of gardening activity for older people, and in particular, to examine the extent to which communal gardening activity on allotment sites may be beneficial to the health and mental wellbeing of older people. | 19 participants who took an active part in communal gardening (65-79 years) (13 men, 6 women) (3 withdrew after 3 months) | England | Project – during a 9 month period the participants gardened on two allotment sites, provided free of charge by Carlisle City Council with the support of a full-time qualified gardener employed by the project. |
| Olsson et al (2013) [33] | To describe how persons with early stage dementia reflect on being outdoors | 11 participants (52-81 years; M = 68 years) in early stage of dementia and living in their own homes (5 women, 6 men) | Sweden | None |
| Raske (2010) [42] | To conduct an in-depth evaluation of the impact of the construction and use of an enabling garden on resident quality of life in a rural nursing home. | 16 residents (65-99 years; M = 81.4 years) (6 men, 10 women) | US | None |
| Rubinstein (1990) [40] | To assess the sense of attachment that older persons may have for their homes/  To examine the relationship of personal identity to environmental meaning in later life | n=7 interviewed but only 3 case studies detailed:  2 men (age 81 and 90), 1 woman (age 75) –AGES CALCULATED FROM BIRTH DATES IN SOME CASES! | Philadelphia, US | None |
| Scott et al (2014) [46] | To explore the reasons for, and importance of, leisure gardening for older Australian adult gardeners residing in the community. Aim was twofold: first, to explore the benefits of regular contact with nature through domestic gardens for Australian community-dwelling older adults; second, to examine the effects of ageing on older adults’ continued pursuit of gardening activities. | 331 participants (60-90 years, mean age 69 years) (? are women, ? are men) | Australia | None |
| Sim et al (2012) [57] | To examine the experiences and effects of relocation as part of a redevelopment of a community housing older people. | 17 participants (residents from a retirement village) (7 men, 10 women) (M = 80.8 years) | UK | None |
| Walker & Hiller (2007) [52] | To explore the social and physical dimensions of neighbourhoods from the perspective of older Australian women and to investigate the variation in these accounts according to whether women lived in areas of higher or lower socioeconomic status | 20 women (75-93 years; M = 82 years) | Australia | None |
| Wang & Glicksman (2013) [48] | To determine the perceived benefits of older adults’ participation in a community gardening programme within low-income senior housing. Specifically, these programmes were community garden of fruits and vegetables tended by older adults living in a senior housing building. | 20 participants (M =71.5 years) (14 women, 6 men) | USA | Project is part of a larger effort, Age-friendly Philadelphia (AfP). GenPhilly, an organisation sponsored by Philadelphia Corporation for Aging, sponsored ‘Germinating Partnerships: Connecting Seniors with Community Gardens’. Three low income housing sites participated in this project. |

**Table S2: Methodological details of included studies**

| **Author(s) and location** | **Approach to Analysis** (what type of analysis) | **Sample**  (how the data were collected) | **Type of sample** | **Theory used**  (drawing on theoretical literature) |
| --- | --- | --- | --- | --- |
| Alves et al (2006) [41] | Coded data independently by two researchers and consensus reached | Semi-structured interviews and photo-elicitation | Purposive | Quality of life (Lawton, 1991) & Day and Cohen (2000) for conceptual framework |
| Bengtsson & Carlsson (2013) [39] | Meaning condensation (Kvale & Brinkmann, 2009) | Semi-structured life-world interview | Purposive | Phenomenological approach – Ulrich’s (1999) supportive garden theory and gerotranscendence (Tornstam, 1997, 2005) |
| Bhatti (2006) [44] | None stated | Written responses to MOS Directives | Purposive | Concept of home and home-making |
| Boyes (2013) [53] | Detailed information emerged inductively | Semi-structured | Purposive | Successful ageing |
| Burton & Sheehan (2010) [34] | Interview data were analysed using both quantitative and qualitative methods. Frequencies and coding used. | Semi-structured interview and photo-elicitation | Purposive | Unspecified |
| Butler & Cohen (2010) [38] | Thematic analysis using a grounded theory approach (study 1); analysed for recurring themes (study 2) | Study 1 – face-to-face interviews  Study 2 – qualitative interviews | Purposive – drawn from Senior Companion Program (Study 1) | No details provided |
| Day (2008) [35] | Thematic analysis | One-to-one semi-structured interviews, two conjoint interviews, group interviews and field observation | Purposive (includes snowball) | No details provided |
| Duggan et al (2008) [49] | Grounded theory approach used to explore emergent themes | Semi-structured interviews | Criterion | Social inclusion (Cantley and Bowes, 2004; Wilkinson, 2002) |
| Durvasula et al (2010) [50] | Major themes derived from interview transcripts | Semi-structured interviews | Criterion | No details provided |
| Edwards et al (2012) [87] | Not stated | Qualitative interviews using open-ended questions | Purposive | No details provided |
| Gibson et al (2007) [36] | Thematic analysis technique drawing upon principles of grounded theory. | Semi-structured interviews; observation | Purposive | No details provided |
| Hassink et al (2010) [37] | Hand-coded interview transcripts, similar and diverging themes emerged from collective review of all coding summaries | Semi-structured interviews | Purposive | No details provided |
| Hawkins et al (2013) [32] | Thematic analysis | Semi-structured interviews | Purposive | Attention Restoration Theory (ART)  Stress Recovery Theory (SRT) |
| Heliker et al (2001) [54] | Content analysis – themes emerged from analysis | Semi-structured interviews | Purposive | Personal meaning framework (Reker & Wong, 1988) |
| Hulko (2014) [88] | No details given | Sharing circles – similar to focus groups involving small-group discussion led by a facilitator but different in that the facilitator mainly listens and observes and cultural protocols are followed | No details given |  |
| Jansen & von Sadovszky (2004) [47] | Content analysis utilizing an empiric-analytic technique (Krippendorf, 1980) | Face-to-face interview | Convenience | Attention Restoration Theory (Kaplan, 1995) |
| Lovering at al (2002) [51] | Notes of observations were transcribed and entered into Ethnograph and open-coded. | Non-participant observation  (In-depth interviews and focus groups with program staff only) | Purposive | No details provided |
| McCaffrey et al (2010) [43] | Thematic analysis | *Stroll for Wellbeing: Garden Walks at the Morikami Museum* – a walking guide and reflective journal. Comprised 12 garden walks, each of which had a theme – awareness, possibility, transition, connection, journey, trust, joy, freedom, forgiveness, reflection, gratitude and fulfilment. Participants wrote on journaling pages after stopping at specific spots and reflecting on the words provided.  Participants were interviewed about the experience of walking in the garden using *Stroll*. | Purposive | Hermeneutic phenomenology and Roger’s Science of Unitary Human Beings (1980) |
| Mapes (2012) [45] | Not stated | Participatory appraisal method - drawings, charts, post-it notes, flip charts, photo and video | Purposive | No details provided |
| Milligan et al (2004) [24] | Analysed using a grounded theory approach | Mixed methodology with key emphasis on ethnography - Focus groups, semi-structured interviews, standard weekly diaries over 9-month period, visual and observational data | Criterion (aged over 65, not mentally confused and had some physical mobility) | Concept of therapeutic landscape |
| Olsson et al (2013) [33] | Qualitative content analysis | Repeated interviews – technique allowed them to tell their own story | Purposive | Social construction theory (Sabat & Collins, 1999; Sabat & Harre, 1992) |
| Raske (2010) [42] | Themes and patterns identified using content analysis. | Semi-structured interviews and photo-elicitation | Convenience | Concept of quality of life (Kane et al, 2003) |
| Rubinstein (1990) [40] | Not stated | Ethnographically-based qualitative interviews, pg.132 & Abstract /  Extended open-ended interviews pg.133 | Convenience sampling based on articulateness rather than for any predisposition to environmental attachment. | No details provided |
| Scott et al (2014) [46] | Statistical analysis of qualitative data – analysed through summative content analysis and Leximancer version 4 text analytics software – uses word association information to elicit concepts, extracting the most important and grouping these according to themes. From a grounded theory approach, Leximancer was used to conduct an automatic analysis of the conceptual content of the participants’ responses to open questions. MORE ON LEXIMANCER? | Survey comprising a series of open questions – participants were provided with lined space to write up to a half page response in the mailed version and equivalent space provided in the online version | Purposive | Theory of successful or positive ageing - Selective Optimization with Compensation (Baltes, 1987) |
| Sim et al (2012) [57] | Thematic analysis – first-order themes and broader second-order themes | Interviews | Purposive | No details provided |
| Walker & Hiller (2007) [52] | Grounded theory approach | In-depth interviews | Criterion | Social capital |
| Wang & Glicksman (2013) [48] | Analysed using open coding, compared and contrasted key words, grouped key words together to form themes | Focus groups | Purposive | Continuity theory (Atchley, 1989); social exchange theory (Blau, 1964); socioemotional selectivity theory (Carstensen et al, 1999) and geotranscendence (Tornstam, 1994, 1999) |

**Table S3.** **Quality Appraisal of Included Studies (Wallace criteria)**

| **Author(s)** | **Is the research question clear?** | **Perspective of author clear?** | **Perspective influenced the study design?** | **Is the study design appropriate?** | **Is the context adequately described?** | **Sample adequate to explore range of subjects/settings?** | **Sample drawn from appropriate population?** | **Data collection adequately described?** | **Data collection rigorously conducted?** | **Data analysis rigorously conducted?** | **Findings substantiated/ limitations considered?** | **Claims to generalizability follow from data?** | **Ethical**  **issues**  **addresse*d*?** |
| --- | --- | --- | --- | --- | --- | --- | --- | --- | --- | --- | --- | --- | --- |
| Alves et al (2006) [41] | Y | Y | Y | Y | Y | Y | Y | Y | Y | CT | Y | N | N |
| Bengtsson & Carlsson (2013) [39] | Y | Y | Y | Y | Y | Y | Y | Y | Y | Y | Y | Y | Y |
| Bhatti (2006) [44] | Y | Y | Y* | Y | Y | CT | CT | N | CT | CT | Y | N | N/A |
| Boyes (2013) [53] | Y | Y | NA | Y | Y | N | Y | Y | CT | N | Y | Y | CT |
| Burton & Sheehan (2010) [34] | Y | N | N | Y | Y | Y | Y | Y | Y | CT | N | Y | Y |
| Butler & Cohen (2010) [38] | Y | Y | Y | Y | Y | Y | Y | Y | Y | Y | N | Y | CT |
| Day (2008) [35] | Y | Y | Y | Y | Y | Y | Y | Y | Y | Y | Y | Y | Y |
| Duggan et al (2008) [49] | Y | Y | Y | Y | Y | Y | Y | Y | Y | CT | Y | Y | Y |
| Durvasula et al (2010) [50] | Y | N | CT | Y | Y | Y | Y | N | N | CT | N | Y | Y |
| Edwards et al (2012) [87] | Y | Y | Y | Y | N | Y | Y | N | CT | CT | N | Y | Y |
| Gibson et al (2007) [36] | Y | Y | Y | Y | Y | Y | Y | Y | Y | Y | N | Y | Y |
| Hassink et al (2010) [37] | Y | Y | CT | Y | Y | Y | Y | Y | Y | N | N | CT | Y |
| Hawkins et al (2013) [32] | Y | Y | Y | Y | Y | Y | Y | Y | Y | Y | Y | Y | Y |
| Heliker et al (2001) [54] | Y | Y | Y | Y | Y | Y | Y | Y | CT | CT | Y | N | Y |
| Hulko (2014) [88] | Y | N | CT | Y | N | Y | Y | CT | CT | CT | Y | N | N |
| Jansen & von Sadovszky (2004) [47] | Y | Y | Y | Y | Y | Y | N | Y | CT | Y | Y | Y | Y |
| Lovering at al (2002) [51] | Y | Y | Y | Y | Y | Y | Y | Y | Y | Y | Y | Y | Y |
| McCaffrey et al (2010) [43] | Y | Y | Y | Y | Y | Y | Y | Y | Y | Y | Y | Y | Y |
| Mapes (2012) [45] | Y | Y | Y | Y | Y | Y | Y | N | CT | CT | N | N | N |
| Milligan et al (2004) [24] | Y | Y | Y | Y | Y | Y | Y | Y | Y | CT | N | Y | N |
| Olsson et al (2013) [33] | Y | Y | Y | Y | Y | Y | CT | Y | Y | Y | Y | N | Y |
| Raske (2010) [42] | Y | Y | Y | Y | Y | Y | Y | Y | N | Y | Y | N | Y |
| Rubinstein (1990) [40] | N | Y | Y | CT | N | CT | CT | N | CT | CT | N | N | N |
| Scott et al (2014) [46] | Y | Y | Y | Y | Y | Y) | Y | Y | CT | CT | Y | Y | N |
| Sim et al (2012) [57] | Y | Y | Y | Y | Y | Y | Y | Y | CT | Y | Y | Y | Y |
| Walker & Hiller (2007) [52] | Y | Y | Y | Y | Y | Y | Y | Y | Y | Y | Y | Y | Y |
| Wang & Glicksman (2013) [48] | Y | Y | Y | Y | Y | Y | Y | CT | CT | Y | Y | Y | N |

*Cannot tell if Bhatti analysed the Directives

**Two studies reported in one article.

**Table S4. Summary of overarching themes**

| **Authors** | **Description from ‘the window’** | **Descriptions emphasising vision** | **Descriptions of ‘Being in Nature’** | | | **Descriptions of ‘Doing in Nature’** | **Barriers** | **Meanings** |
| --- | --- | --- | --- | --- | --- | --- | --- | --- |
|  |  |  | **Fresh air** | **Peace** | **Social** |  |  |  |
| Alves et al (2006) [41] | **✓** |  |  |  | **✓** |  |  |  |
| Bengtsson & Carlsson (2013) [39] | **✓** | **✓** | **✓** |  | **✓** |  | **✓** | **✓** |
| Bhatti (2006) [44] |  | **✓** |  |  |  |  | **✓** |  |
| Boyes (2013) [53] |  |  |  |  |  | **✓** |  |  |
| Burton & Sheehan (2010) [34] | **✓** |  |  |  |  |  | **✓** |  |
| Butler & Cohen (2010) [38] | **✓** | **✓** | **✓** | **✓** |  | **✓** | **✓** |  |
| Day (2008) [35] | **✓** | **✓** | **✓** | **✓** |  | **✓** |  |  |
| Duggan et al (2008) [49] |  |  | **✓** |  | **✓** |  | **✓** |  |
| Durvasula et al (2010) [50] |  |  | **✓** |  | **✓** |  | **✓** |  |
| Edwards et al (2012) [87] |  |  |  |  |  |  |  | **✓** |
| Gibson et al (2007) [36] | **✓** |  | **✓** |  |  | **✓** | **✓** |  |
| Hassink et al (2010) [37] |  | **✓** |  |  |  |  |  |  |
| Hawkins et al (2013) [32] |  |  | **✓** | **✓** |  | **✓** |  |  |
| Heliker et al (2001) [54] |  |  |  |  |  | **✓** |  | **✓** |
| Hulko (2014) [88] |  |  |  |  |  |  |  | **✓** |
| Jansen & von Sadovszky (2004) [47] |  | **✓** |  |  |  | **✓** |  | **✓** |
| Lovering at al (2002) [51] |  |  |  |  | **✓** |  | **✓** |  |
| McCaffrey et al (2010) [43] |  | **✓** |  | **✓** |  | **✓** |  | **✓** |
| Mapes (2012) [45] |  | **✓** |  |  | **✓** |  |  |  |
| Milligan et al (2004) [24] | **✓** | **✓** |  | **✓** |  | **✓** |  |  |
| Olsson et al (2013) [33] |  |  | **✓** |  | **✓** |  | **✓** |  |
| Raske (2010) [42] |  | **✓** |  | **✓** | **✓** | **✓** |  | **✓** |
| Rubinstein (1990) [40] | **✓** |  |  |  |  |  |  | **✓** |
| Scott et al (2014) [46] |  | **✓** |  | **✓** |  | **✓** | **✓** | **✓** |
| Sim et al (2012) [57] |  |  |  |  |  |  | **✓** |  |
| Walker & Hiller (2007) [52] |  |  |  | **✓** |  |  |  |  |
| Wang & Glicksman (2013) [48] |  | **✓** |  | **✓** |  | **✓** |  |  |
